# Integrating community children’s nursing in urgent and emergency care: a qualitative comparison of two teams in North West England

**DOI:** 10.1186/1471-2431-12-101

**Published:** 2012-07-16

**Authors:** Richard G Kyle, Michele Banks, Susan Kirk, Peter Powell, Peter Callery

**Affiliations:** 1 School of Nursing, Midwifery and Health, University of Stirling (Highland Campus), Centre for Health Science, Old Perth Road, Inverness, IV2 3JH, UK; 2 School of Nursing, Midwifery and Social Work, The University of Manchester, Manchester Academic Health Science Centre, Jean McFarlane Building, Oxford Road, Manchester, M13 9PL, UK; 3 West Suffolk Hospital, Bury St , Edmunds, UK

## Abstract

**Background:**

Despite the policy principle that “children are best cared for at home whenever possible” children continue to have high rates of emergency department (ED) attendance and emergency hospital admission. Community Children’s Nursing Teams (CCNTs) can care for acutely ill children at home but their potential to provide an alternative to ED attendance and hospitalisation depends on effective integration with other services in the urgent care system, such as EDs and Observation and Assessment Units (OAUs). Although challenges of integrating CCNTs have been identified, there has been no comparative assessment of the factors that facilitate or hinder integration of care of acutely ill children by CCNTs with the urgent care system. The aim of this study was to identify enablers and barriers to integration of CCNTs with urgent and emergency care.

**Methods:**

Comparative case studies were conducted of two CCNTs serving Primary Care Trusts in North West England. Twenty-two health professionals including CCNT managers and staff; paediatricians; nurses; children’s ward, ED and OAU staff; commissioners of children’s services; GPs and primary care staff were interviewed between June 2009 and February 2010. Qualitative data were analysed thematically using the Framework approach.

**Results:**

Barriers to integration included paediatricians’ perceived lack of ownership of the CCNT, poor communication between consultants and community children’s nurses (CCNs), and weak personal relationships. This prevented early referral to the CCNT as an alternative to hospital care. Enablers of integration included co-location and rotation of CCNs through urgent care settings including OAUs and EDs. This enabled nurses to develop skills, make decisions about referral to home care and gain the confidence of referring clinicians.

**Conclusions:**

Integration of CCNTs at multiple points in the urgent care system is required in order to provide an alternative to inappropriate ED attendances and emergency admission. The principal enablers and barriers are both aspects of normative integration, which involves shared understanding of the contribution of CCNTs and trusting relationships between practitioners. Co-location and rotation of CCNs through acute services can promote integration and appropriate referrals to CCNTs to support families to care for children at home.

## Background

Despite the policy principle that “children are best cared for at home whenever possible” [1] children continue to have high rates of emergency department (ED) attendance and emergency hospital admission [2-4]. Community Children’s Nursing Teams (CCNTs) can care for acutely ill children at home [5] but their potential to provide an alternative to ED attendance and hospitalisation depends on effective integration with other services in the urgent care system such as EDs and Observation and Assessment Units (OAUs) [6]. There is substantial variation between CCNTs in their extent of integration with the urgent care system as indicated by routinely collected referral source data and parent-reported average number of services used by their acutely ill child prior to CCNT referral [7]**.** Challenges of integrating CCNTs have also been identified relating to referral practice, service transition, inter-professional communication and joint-working [8]. However, there has been no comparative assessment of the factors that facilitate or hinder integration of care of acutely ill children by CCNTs with the urgent care system.

The aim of the study was to identify enablers and barriers to the integration of CCNTs with the urgent care system that includes General Practitioners (GPs), EDs, OAUs and paediatric wards.

## Methods

### Design

Case studies were conducted of two CCNTs serving two Primary Care Trusts (PCTs) in North West England. PCTs in England performed three main functions: 1) improving the health and wellbeing of the population they served (average 330,000) through reduction of health inequalities, health protection and emergency planning, often in partnership with Local Authorities (70% of which had coterminous boundaries); 2) commissioning and designing primary and secondary care services, including mental health, GP, dental and pharmacy services, as well as screening programmes and patient transport; 3) investing in staff development, capital infrastructure and information technology. At the time of the study, there were 152 PCTs across England which controlled 85% of the total National Health Service (NHS) budget [9].

Purposive sampling was conducted to ensure selected CCNTs had different organisational and population characteristics as well as varying levels of integration with the urgent care system as indicated by referral source (see Table 1).

**Table 1:** Case study population and service characteristics

		**Case study**		
**PCT Population**		**A**		**B**
Population^1^	<15 years old, n (%)	34,300 (18.7)		45,100 (18.1)
Deprivation^2^		17.9		34.5
Child Well-being^3^		7.5		20.4
**Local Healthcare Economy**				
Services				
Hospital		District General		District General
ED		Yes		Yes
OAU		No		Yes
Walk-in Centre (n)		Yes (2)		No
Emergency Admission Rate at Local Hospital^‡4^		42.3		52.3
ED attendance rate at Local Hospital^‡5^		376.2		385.1
GPs per 100,000 children <15^6^		306.4		295.2
**CCNT**				
Base (Organisation)		Community (PCT)		Hospital (Acute Trust)
Number of years established at beginning of study		3		14
Disease focus		Acute and Chronic		Acute (and End of Life)
Referrals^7^	n	923		3,024
	Source (%)	Ward	77.0	35.2
		GP	7.3	16.0
		Walk-in-Centre	5.2	-
		School Nurse/Health Visitor/Midwife	5.0	-
		Parent/Carer	2.1	1.5
		Other CCNTs	1.7	-
		ED	1.1	26.0
		OAU	-	15.8
		GP out-of-hours	0.2	-
		Out-patients	-	2.1
		Other	0.4	3.4
Workforce^8^ FTE	n		13.8	14.4
	per 1,000 children <15		0.40	0.32
Hours of operation		Mon to Fri 08:00 to 20:00;		Mon to Sun 08:00 to 20:00
		Sat/Sun/Bank Holidays 08:00 to 18:00		
CCNT referral rate per 1,000 children <15^9^		26.9		67.1

^‡^ Local Hospital is defined as the hospital to which the greatest percentage of children resident in the PCT attend.

^1^ Office for National Statistics (ONS) mid-year estimates 2008.

^2^ Percentage of people living in the most deprived quintile of the Index of Multiple Deprivation 2007 (England average: 19.9%) (Source: APHO and DH Health Profile 2009).

^3^ Percentage of Lower Super Output Areas in lowest quintile of the national distribution (Source: Local Index of Child Well-being 2009).

^4^ Emergency Admission Rate for three commonest medical presentations at EDs (i.e., breathing difficulty, feverish illness, diarrhoea) per 1,000 children aged 0–14 resident in the study area (Source: Hospital Episode Statistics 2006/07).

^5^ ED attendance rate per 1,000 children aged 0–14 registered with a GP in the study area (Source: North West Strategic Health Authority Tactical Information Service 2007/08).

^6^ GPs per 100,000 children aged 0–14 (Source: The Information Centre for Health and Social Care 2009).

^7^ Annual Referrals (Source: CCNT routinely collected data 2009).

^8^ Full-time Equivalent workforce (Source: Service A, December 2009; Service B, March 2010).

^9^ CCNT referral rate per 1,000 children aged 0–14 (Source: CCNT routinely collected data 2009; ONS mid-year estimates 2009).

A qualitative approach was adopted to elicit the views of healthcare professionals on the perceived barriers and enablers of integration of the CCNT with the local urgent care system.

### Sample

Twenty-two health professionals were interviewed across the two case studies between June 2009 and February 2010 (10 in case study A and 12 in case study B) (Table 2). Individuals were purposively sampled to provide a diverse range of professional perspectives from services in the local healthcare economy and included: consultant paediatricians; children’s ward, ED and OAU managers; commissioners of children’s services; specialist nurses and paediatric liaison health visitors; GPs; clerical and clinical CCNT staff, including student and staff nurses, and managers. Key stakeholders from different organisational standpoints and levels of seniority within each case study site were identified as potential interviewees and approached by researchers (RGK, MB) by telephone or email.

**Table 2:** Interviewees’ organisational location

	**Case study**		**Total**
**Organisation**	**A**	**B**	
Community Children’s Nursing Team (CCNT)	4	6	**10**
Hospital	3	3	**6**
Other (i.e., commissioners, primary care)	3	3	**6**
Total	**10**	**12**	**22**

### Data collection

Semi-structured face-to-face interviews were conducted with participants. Each interview lasted around an hour and was conducted at the interviewee’s workplace. Interviews were structured by the following topic guide:

· Individual’s roles, responsibilities and professional background;

· Views around the current and future role of the CCNT;

· Perceived impact of the development of CCN services on roles, relationships, skills, training needs and the local healthcare economy;

· Impact of past, current and future service reconfiguration;

· Impact of the extension of the CCNT’s role in acute care on work with children and young people with long-term conditions;

· Referrals to other agencies such as social care, child protection and education.

The form of the interviews was conversational to allow participants to express and explain their views. The topic guide was tailored to each interviewee and additional questions were included to address specific aspects of each individual’s role, organisational location or level of engagement with the CCNT. Interviewees were also encouraged to raise further issues that they considered relevant from their own perspective. Data collection was conducted by two researchers (RGK, MB). Adherence to the topic guide during interviews was ensured through discussion between researchers following each interview.

### Data analysis

Interviews were digitally audio recorded and transcribed verbatim. Data were managed using NVivo (Version 8). Thematic analysis was conducted using principles and procedures of the Framework approach [10]: i.e., (1) familiarisation with the data through the independent reading of each transcript by two co-authors; (2) development of the thematic framework at two ‘data workshops’ involving four co-authors (RGK, MB, SK, PC); (3) indexing of the data corpus by two co-authors (RGK, MB). To examine inter-coder reliability a 5% sample of indexed data was cross-checked. This revealed no substantial differences in the application of the thematic framework between coders; hence no further data were cross-checked. At each stage, analysis was guided by core research questions including the question reported here: what facilitates and inhibits integration of CCNTs within the urgent care system?

### Ethical approval

Ethical approval for the study was obtained from university and local NHS research ethics committees. Interviewees provided informed written consent. In order to maintain confidentiality individuals are identified throughout this paper by a case study letter (i.e., A or B), interviewee number and their role, designated as ‘Paediatrician’, ‘CCN’ (Community Children’s Nurse) or ‘Manager’ (e.g., B6: CCN).

## Results

### Service profiles

Both the case study CCNTs were members of a NHS network that coordinated recruitment and training of CCNs. This enabled the development of common protocols for use in different settings, for example for nurses to give intravenous medication in different community or hospital NHS Trusts. However the case study CCNTs differed in terms of service design, workforce, operation, and the nature and extent of relationships with urgent care services in the locality (Table 1).

### Case study A

#### Service organisation

The CCNT in case study A was mainly a follow-up service from the ward, which was the source of 77% of its referrals. It had little integration with the ED (1.1% of referrals) or GPs (7.3%), despite being based in a community setting (Table 1). Parents reported using an average of three services prior to CCNT referral [7].

#### Service mission

Professionals in case study A reported that the purpose of the CCNT was variously to: (1) prevent ED attendance and emergency admission; (2) facilitate early discharge; (3) encourage direct GP referrals by acting as a *“stop-gap”* (A4: CCN) to reduce immediate reliance on secondary care settings. However, there was little evidence that this service mission was widely shared among these professionals. This was demonstrated through: (1) disagreement between senior clinicians around the extent to which the CCNT had changed discharge practice;

“Perhaps [discharge] is a little bit earlier because [the CCNT] now have extended their service over the weekend and so on, but I wouldn’t say it’s vastly different” (A10: Paediatrician).

*“To some extent it definitely had impact, because my threshold for sending children has dropped. I’ve discharged much earlier, for example, meningitis on day three they go home now.” (A7: Paediatrician)*"

(2) discussion about the mandated nature of the CCNT;

"*“It’s not optional whether we have one or not, because we signed up to the joint committee of PCTs, we have to have a CCNT. […] I could envisage different models which provided the same benefits, but […] would not necessarily need to be a CCNT.” (A9: Manager)*"

(3) managers’ and clinicians’ envisioning of alternative service models to deliver these three aims.

*“I’d love to do a clinic in [the] town centre every day, afternoon I can do it, [a] clinic so GPs can tell the parents ‘look, this afternoon there’s a paediatric clinic’ and I’ll ask different consultants doing paediatric support and that’s the way […] we can diagnose conditions early and avoid admission, unnecessary admission and parents can be reassured. So what I can do, and I can then tell community nurses ‘look I’ll send this child, I’m not worried about this child, these are the things I’ll advise, you look after, and I’ll take it up with the GP”. (A7: Paediatrician)*"

Thus some stakeholders reported only limited professional investment in the CCNT.

### Case study B

#### Service organisation

In contrast the CCNT in case study B was integrated at multiple points in the urgent care pathway, receiving 26% of its referrals from the ED, 15.8% from OAU, 16% from GPs, although the largest proportion (35.2%) were from the ward in its hospital base (Table 1). Parents reported using an average of two services prior to CCNT referral [7].

#### Service mission

The same tripartite service mission was expressed by interviewees in case study B: (1) prevent ED attendances and emergency admission; (2) enable early discharge; (3) encourage direct GP referral. However, in contrast to case study A this mission was widely shared among professionals. Interviewees agreed that the CCNT in case study B was a *“hub”* (B1: Paediatrician) and a *“link between [the OAU and ED]”* (B2: Paediatrician) that *“jump[s] in [to] integrate [a child] back into the system”* (B2: Paediatrician). Importantly, this mission had been carefully constructed and cultivated through considerable financial and human resource investment in the design of the acute and urgent care pathway, for instance through appointment of consultants with areas of expertise that complemented the ambulatory care model. Further, in contrast to case study A, professionals identified a continuing role for the CCNT in these urgent care pathways.

*“That’s where I think the community nursing teams are more actively involved at the moment, with children who are admitted to OAU and who are being discharged to the community from there. I want to bring that process further down here and take that step from the ED rather than OAU.” (B2: Paediatrician)*"

### Summary

Case study A therefore provides an example of a CCNT that was weakly integrated with the urgent care system, whereas case study B exemplified a CCNT that exhibited greater integration. Barriers and enablers to integration with urgent care services were identified in case studies A and B, respectively, and are reported in turn in the remainder of this section. Because the majority of CCNT referrals came from hospital wards and EDs (Table 1), the findings pertain primarily to CCNT integration in secondary care settings.

### Barriers to integration of CCNT with urgent care

Although there was evidence of integration within case study A, principally as a result of nurse-to-nurse communication and referral, comments by paediatricians demonstrated the existence of three inter-related barriers to integration that revolved around consultant-CCN relationships and communication: (1) lack of ownership of the current CCNT service model; (2) poor communication with the CCNT; (3) absence of personal relationships with CCNs.

#### Lack of ownership over CCNT

Concerns about ownership centred on the organisational location of the CCNT in the PCT rather than in their acute Trust.

*“You know, when I had this nurse and she was appointed by me, an asthma nurse, 35*% *of my patients I never used to see them, she would see them. They are much better. My role is to diagnose.” (A7: Paediatrician)*"

*“My perception is they should be based in the same organisation and should be working very closely with the consultant rather than working in isolation. […] It would have been so much better if they were based under me and were regularly in the ward, they would have seen what the current policies [were] and taken those out, communications would have been more direct and it would have worked much better.” (A10: Paediatrician)*"

#### Poor communication with CCNT

Frustration with poor communication revealed an underlying anxiety that CCNs were an unknown quantity outside consultant control.

*“They’re doing their own thing, get no communication whatsoever unless I have requested [it]. Definitely there’s a good habit now, people use the paediatric nurses but what they do only they know, no communication back.” (A10: Paediatrician)*"

*“It started back in [the early-1990s], we started with one nurse who was extremely good, who was based within the same management as the hospital and communication was so much easier.” (A10: Paediatrician)*"

#### Absence of personal relationships with CCNs

Weak personal relationships generated anxiety around clear lines of clinical and managerial responsibility. This was evident in consultants’ unwillingness to delegate the personal trust invested in them by parents to CCNs.

*“The way I work with my team is I know them very well, I know whom to trust, I know whom to contact and unless you’ve got that kind of personal relationship you cannot. […] I don’t know the community nurses, they’re nice, I’ve met them in a couple of meetings but I don’t know which person I can discharge to whom and that inhibits me because I know Mum very well, Mum has faith in me, so she expects me to look after her child. I can only transfer it while that relationship [exists].” (A7: Paediatrician)*"

### Consequences for patient care

Combined, these barriers had three notable consequences for patient care: (1) potential for early discharge from the ward was limited; (2) efforts of NHS staff were duplicated with associated increased costs to families; (3) potential for inequities in access to CCNT care.

#### Limited potential for early ward discharge

Increased potential for early discharge from the ward to the CCNT was reported to be contingent upon improved personal relationships. One individual estimated that the current impact of the CCNT on early discharge was *“probably 30*%*”* and that this proportion could increase by a further *“60-70*%*”* through better working relationships (A7: Paediatrician).

*“If I have a very good relationship with community nurses I would probably discharge 90*% *of children from the ward.” (A7: Paediatrician)*"

*“I don’t think that all paediatricians, certainly not all ED consultants and people have got confidence or are clear about what they can discharge back out to the care of the team and the competency of the team, which hugely undermines the process potentially.” (A9: Manager)*"

#### Duplication of effort and increased costs

Lack of trust in the CCNT’s capability resulted in duplication of effort as some children were brought back to the hospital as ward attenders even after they had been referred to CCNT care.

*“What they’re also doing at [the hospital] as well is the children that are borderline for admission, they’re not referring them out to us, they’re bringing them back as ward attenders the next day to be checked out. So, I have got to get in and influence that system as well because we’ve been having a couple of children that have been referred out and they’re going back as ward attenders as well. Well, if you’re going to refer them to us, leave it to us, and we’ll contact you, but it’s historic.” (A1: Manager)*"

*“At the moment what we are doing is we’re attending on the ward on a regular basis, maintaining those links, and although we felt that we were getting the right referrals from this information coming through about children being brought back to the ward as attenders it would appear actually we’re getting not necessarily the right children […] which now is going to bring us back into another discussion about have we got that relationship as good as we thought we had, or is it a case of doctors having more power than the nurses?” (A8: Manager)*"

"This duplication had the potential to increase financial costs to the NHS and also to families. Avoidable returns to hospital for illness that may be managed in the community are also potentially disruptive for children and their families."

#### Potential for inequities in access to CCNT

CCNT referral was also not routinely discussed by paediatricians with parents at the point of discharge. Due to their lack of personal relationship with CCNs consultants delegated responsibility to initiate CCNT referral to the discretion of junior clinicians, with the potential for referral decisions to be inconsistent.

*“At present my worry is I don’t know many of the community nurses, I don’t have that relationship where I can say, okay, I know you very well and I can trust you, you can look after. So what happens is I leave it to the nursing staff on the ward to decide because they have got better relationships because they talk to them.” (A7: Paediatrician)*"

Paediatricians proposed integrating CCNs into the paediatric team as an alternative model. It was suggested that control by the paediatricians could address concerns about the extent and timeliness of communications and enhance personal and professional relationships to ensure confident delegation of parental trust.

*“Ideally I would like them to be based in where I am and they should be going, there should be a regular end of clinic chat with the community paediatric nurse with every consultant and then they can take the work out in the community.” (A10: Paediatrician)*"

The repeated use of terms such as *“taking the work out into the community”* (A10: Paediatrician) suggested that paediatricians viewed the role of CCNTs through a secondary care lens as an extension of (their) hospital care and expertise at home.

### Enablers of integration of CCNT with urgent care

Interviewees in case study B identified three core components of service design that enabled integration: (1) co-location of the CCNT with acute services; (2) CCNT/OAU rotation; (3) embedded senior paediatric nurses in the ED. Each component was observed to strengthen personal and professional relationships between the CCNT and urgent care.

#### Co-location

Interviewees reported that co-location of the CCNT base in an acute care setting (for example near the OAU, ED and the paediatric ward) delivered a number of benefits including the ability to borrow supplies, obtain emergency prescriptions or effect introductions with anxious parents, and streamlining of safeguarding processes. The main advantage of co-location was that clear communication channels were opened with colleagues and particularly with paediatricians who often proved elusive.

*“If there is anything that we ever need or there’s anything we need to discuss we know that we can just walk down the ward and discuss it there and then.” (B6: CCN)*"

*“I have very good links with the consultants and staff so can get decisions made a lot quicker.” (B14: CCN)*"

*“It’s quick, it’s direct. It takes them three minutes to walk round, they can knock on my door and they can get an answer. So it does mean we can be more productive in that sense and everyone’s got an awareness.” (B1: Paediatrician)*"

CCNs and consultants also shared a belief that geographic proximity increased productivity through quicker and more confident decision-making.

*“The team here have better relationships with the medical team and […] I think they feel more secure and more able to make better decisions within the community because they’ve got that medical support.” (B14: CCN)*"

*“I suppose for us I feel that because we’re embedded with the paediatric services and it’s allowed us to as well be really quite autonomous in the way that we work. We’re very, very familiar with the protocols, the guidelines, the evidence base that the unit as a whole is working within and I think it’s important that parents get the same message. I don’t know if we would be able to work quite so efficiently if we were having to take our help from GPs and maybe other professionals who maybe could pose more challenges to us.” (B9: Manager)*"

*“Whereas with anything we need to ask about bloods or anything like that, we can just pop on the Ward, and there'll generally be a doctor that will, and they respond really well to us, because they know us all anyway. So we work really closely with them, you can always, all the consultants know everybody on the team, so they're quite good at, you know, they know us, they'll refer stuff to us, and we'll ask for them to review people in the clinic, and they'll pop them in on a clinic day." (B6: CCN)*"

Indeed, co-location ensured that the CCNT was an integral part of the urgent care pathway and that it acted as a *“hub”* (B1: Paediatrician) at system-level. The CCNT was observed to perform a co-ordination role, linking various services and professionals

Interviewer: “It's interesting that you talked about the CCNT as almost like a hub. Is that how you envisage it?

Interviewee: well I think we're all feeding in, but actually they, they can link us all up together,

Interviewer: Yeah, so really, like you say, that's the vehicle to get the coordination, it's interesting,

Interviewee: Mm, well they're co-located with us actually, if we're going to do ambulatory, it's probably got to be centred around them” (B1: Paediatrician)

Moreover, the effectiveness of this co-ordination role was reported to be a consequence of clinicians’ confidence in CCNs skills and expertise as autonomous practitioners and CCNs confidence in their own ability and autonomy.

*“CCNs can go down, actually look at the child, and if, you know, if s/he definitely was needing any further interventions, they can bring a child to OAU. So they are a link between those two areas as well, and if the child was deteriorating at a faster rate then they can come back to the ED as well. So I think that is a very, very important link, that a person on a team is available on the background to follow these children up, to jump in, integrate him back to the system, which would have been very delayed and fragmented for a child who's been going off, when he was discharged from the ED or OAU, and the parents are anxious, and they don't get appropriate help at the appropriate time. And again that small window there if the child is very unwell, you know, so it's a very, very important role they've got." (B2: Paediatrician)*"

*“A very speedy communication process as well, you know, and again that must reassure the parents really, that they can, they can see that those arrangements are in place, like for instance, when you know you can go in, you can say, well we, don't worry, if there is a problem with your child I can directly re-admit your child to a Children's ward, I have that autonomy.” (B13: CCN)*"

#### CCNT/OAU rotation

Rotation between the CCNT and OAU was instigated by two nurses through a job share arrangement and was perceived to: enable continuity of care and potentially reduce visit times; streamline and personalise communication as nurses attained ‘insider’ status in each team; increase understanding around nursing roles and enable nurses to be used as a *“resource”* (B4: CCN); encourage learning and professional development.

Interviewee: What we try to do as best as we can is, if we've worked, say I'm in the OAU today now 2 'til 10, and I'm in the CCNT tomorrow 8 'til 3, any patients I see in the OAU today I'll put my name down to see them then tomorrow at home,

Interviewer: Right, so there's that continuity.

Interviewee: Yeah, so you get a very good continuity now of service, and by doing that, it actually reduces visit times down then for me for tomorrow, because I've already taken the history in OAU today. I know all about what the doctor's said, I know all about what medication we've put them on, I'm literally going tomorrow just to, to check up on them really, so that it, it kind of, it works in everybody's favour." (B4: CCN)

*“We always make sure one of us is at […] the meetings and then it’s to feed back really to the other team any issues that have arisen, there’s a few queries around this, what do you think about this so I can feed back at the next meeting.” (B4: CCN)*"

Visibility of CCNs in urgent care settings and frequent demonstration of skills fostered a mutual appreciation and respect for skills and experience, highlighting that working in the community was a source of expertise rather than just a different location for the application of hospital care.

*“The more junior staff nurses on the ward area you’ll often find they’ll come down to the OAU and say; ‘Could I just ask you about this one? I’m not sure whether it’s something that could be doing in the community’. […] you could have people ringing you up to say; ‘Not sure whether to send this one back in [to hospital] or not, what would they do differently on the OAU that we can’t do at home?’” (B4: CCN)*"

*“In the very early days there was a lot of negativity really between the CCNT and the OAU just because neither of those areas can understand the other one’s roles, you just can’t unless you’ve worked in both areas you cannot fully appreciate the challenges in each other’s roles. So I think by doing the shared job now it’s just made relations between the two areas absolutely 100*% *improved.” (B4: CCN)*"

#### Embedded senior paediatric nurses

Embedded senior staff in emergency care settings, particularly Advanced Paediatric Nurse Practitioners (APNPs) in the ED, were perceived to deliver considerable benefits. APNPs were regarded as *“gate-keepers”* (B9: Manager). By positioning these professionals *“at the front door”* (B1: Paediatrician) children could be quickly referred to the most appropriate service, such as the CCNT or OAU, potentially reducing unnecessary ward admissions and shortening the *“patient pathway”* (B1: Paediatrician).

*“In the ideal world really some more closer ties with the ED to try and turn them away before they actually get to the ward they should be turned away at the front door with continuity of care, the same nursing staff over the next day or two.” (B1: Paediatrician).*"

Advanced skills and *“bravery”* (B9: Manager) of autonomous decision-making were also considered to encourage learning and professional development among colleagues through *“role-modelling”* (B9: Manager).

*“Role-modelling, and looking at ways of working, they’re going to have it demonstrated to them all the time, demonstrating on bravery of decisions, which sometimes they’re not always able to make.” (B9: Manager)*"

#### Potential barriers to closer integration

Professionals identified two potential barriers to closer integration in case study B: (1) role definition; (2) harmonisation of clinical governance protocols.

#### Role definition

Nurses required additional training to work in both community and hospital settings and they needed to practice skills regularly in order to retain competence and confidence. There could be problems of specifying clear roles for CCNs/APNPs when working in the ED, particularly during busy pressurised periods when there was increased potential for them to be used inappropriately and inefficiently.

*“We have two advanced nurses that have just gone through the training, obviously what we want, eventually, is advanced nurses that can cover 24/7 in ED, we’re not anywhere near that yet, however they’ve completed the competencies, they’re consolidating and they’re about, one of them is in, mainly based in ED now.” (B13: Manager).*"

*“Our advanced nurses are learning from our consultants and our medics, the medical workforce […i]t’s an exciting role, but it’s also quite a scary role, when you’re out there on your own to start with making decisions, and it’s about that, if you talk to them it’s about “Ooh, I’ve done A, B and C, but I just wanted to check with somebody that that was right, it was the right decision”, until you grow in confidence.” (B13: Manager).*"

#### Harmonisation of clinical governance protocols

Working across settings also highlighted the need for harmonisation of clinical governance processes, which it was noted could be facilitated by co-location.

*“I think some of the governance issues definitely in the ED department here have not been addressed and they’ve not been resolved.” (B13: Manager)*"

## Discussion

Several definitions of integration have been proposed in the research literature, although opinion converges around a five-fold typology: (1) Administrative (aligning back-office functions and financial systems); (2) Systemic (co-ordinating and aligning policies, rules and regulatory frameworks); (3) Organisational (co-ordinating structures, governance systems and relationships); (4) Clinical (co-ordinating patient care through shared guidelines and protocols); (5) Normative (developing shared values, culture and vision across organisations and professionals) [11]. Our study identified the importance of normative integration to the effective integration of CCNTs in the urgent care system. Normative integration involves “developing common integration goals, identifying and addressing communication gaps, building clinical relationships and trust” [11]. A central issue that emerged in both case studies was the need for shared vision of how the CCNT could contribute to care and understanding of the capabilities of CCNs. In case study B participants attributed successful integration to trusting relationships that enabled paediatricians, ED clinicians and GPs to refer children for care by CCNTs. Personal and professional trust enabled the safe and effective delegation of responsibility and ultimately risk from paediatricians to CCNs. This was facilitated through the geographical proximity of the CCNT to urgent care settings through co-location and relational proximity through rotation and embedded advanced practitioners. APNPs had the support of consultant paediatricians which empowered them to work autonomously and to make clear decisions to care for children at home rather than in hospital. In contrast participants in case study A reported that lack of trust in, and communication with, CCNTs inhibited referral. Paediatricians’ perceived CCNs to be an extension of hospital care in the community and were generally unwilling to recognise the CCNT as an independent service with its own skills and expertise to assess whether children could continue with home care or required hospital admission.

It could be that differences in service organisation contributed to variation in reported experiences of integration between the two case studies. More specifically, opportunities to implement certain aspects of service design that were found to be facilitative of integration may have been limited in case study A because the CCNT was based in a community setting. This may mean that potential benefits of geographical and relational proximity identified in case study B could not accrue, perhaps through decreased visibility of CCNs [8]. The combined acute and chronic case-load in CCNT A may also contribute to consultant paediatricians’ confusion around the role, and concerns about the competency, of CCNs in the management of children with acute illnesses. In addition, CCNT B had been established for considerably longer (14 years) than CCNT A (3 years) and it is possible that relational barriers similar to those evident in CCNT A may also have been encountered and overcome in CCNT B at an earlier stage of its development.

However, cultural change can be as important as organisational structures to successful integration of health care services [12]. An ethos of shared values and commitment can enable trust and collaboration to develop [13]. This normative integration was a necessary condition for effective referral to CCNTs at a sufficiently early point in children’s contact with the urgent care system to avoid hospitalisation. There was shared understanding of the purpose and potential contribution of CCNTs and trust in individual CCNs in case study B. This provided the basis for developing clinical integration (i.e., “integrating patient care within a single process” [11]), including procedures for referral to CCNTs from various points in the urgent care system, including GPs, EDs, and OAUs, as well as the paediatric ward. The lack of communication and personal relationships in case study A prevented the confident delegation of clinical risk and parental trust to CCNs. There was only weak clinical integration between the CCNT and the wider urgent care system which limited opportunities for early referral and avoidance of hospitalisation. Paediatricians expressed a desire for greater organisational integration to address their lack of ownership and control over the CCNT. Paediatricians may feel more confident to delegate the trust and clinical responsibility invested in them by parents to CCNs if they have ownership of the CCNT as part of their team. However organisational integration alone cannot make the links with the various services in the urgent care system required for early intervention by CCNTs to prevent admission rather than just to follow up children after discharge.

Lack of shared understanding of purpose and effective procedures for referral have been identified as key problems in integration of primary care practitioners in emergency departments [14,15]. It is important to “break down the barriers between primary care and emergency care clinicians” and to develop strong working relationships and build mutual respect, clarity about the strengths of different professionals, how they are best deployed and expectations of each group [16]. The importance of personal relationships is highlighted by reports that regular use of the same individuals leads “to more coherent and higher quality clinical decisions.” [16]. Thus normative integration has been identified as a key requirement for effective contribution in emergency care by primary care practitioners as well as the CCNs in this study.

Normative and clinical integration in case study B were facilitated by rotation into acute settings which nurses described as facilitating development of observation and assessment skills to detect changes in children’s condition. This gave nurses confidence to work autonomously in the community. Rotation between community and acute settings also enabled CCNs to demonstrate their skills in the ED and OAU settings and the confidence this gave clinicians in CCNs’ competence enabled early referral to the CCNT. Nurses also described how their presence in the ED or OAU could act as a prompt to remind professionals to refer children to CCNT care. This suggests that CCNs’ experience of taking responsibility for care of children in the community – and in so doing managing risk – enabled them to identify opportunities for referral to home care of children who could otherwise have been cared for in hospital. This experience of autonomous decision-making can therefore enable appropriate referrals that structured referral criteria and process alone may not achieve. This suggests that there is a balance to be struck between over- and under-defined CCNT referral criteria. There must be sufficient flexibility in the referral process to allow individuals to use discretion to make judgements. This requires that nurses have sufficient training and experience to exercise discretion, and have the authority to take decisions, which requires trusting professional relationships between providers across the urgent care system.

Our findings suggest that it is important that current reforms to commissioning in the NHS in England [17] which may alter the interface between CCNTs and urgent care services, such as paediatric EDs and OAUs, do not ‘structure out’ opportunities for nursing staff to develop and use professional discretion. Caution should also be exercised in service reconfigurations to avoid unnecessarily severing trusting relationships between professionals which take a long time to develop [18]. Local emergency and urgent care networks may therefore play a central role in managing future change to minimise the potential negative relational impact of reform or reconfiguration on existing examples of effective normative integration. Efforts to encourage or preserve normative integration, such as joint involvement in service (re)design, are important to support the development of local processes and protocols to facilitate clinical integration of CCNTs across the urgent care system. In turn this may maximise potential benefits to children and families in terms of reduced psychosocial and financial burden of potentially avoidable ED attendances and emergency admissions, and realise potential efficiency savings for the NHS [18].

### Strengths and limitations

To our knowledge this is the first comparative study of the enablers and barriers to the integration of CCNTs in urgent care. Previous research has principally been composed of evaluations of single CCNTs [19] with little consideration of how services integrated with the wider healthcare economy, so that the literature provides limited evidence about best practice in organisation of services [20]. A particular strength of the comparative case study approach is that complex phenomena can be understood [21] by drawing on multiple perspectives, in this case of professionals in different services, organisations and roles across the urgent care system. Thus, although comparison of only two case studies may limit the generalisability of these findings, because the case study approach takes into account the particular case study contexts in order to reach conclusions, more general lessons to inform practice and policy development can be drawn.

## Conclusions

This comparative study of CCNTs highlights the importance of shared understanding of their contribution to children’s urgent and emergency care pathways and of trusting relationships between practitioners. These are features of normative integration, which is necessary for the development of clinical integration and referral to care by CCNTs which avoids hospitalisation. While organisational integration may be achievable relatively quickly, the development of trusting relationships and consistent clinical protocols can be time consuming. There is a need for longitudinal research to identify how normative and clinical integration can be expedited so that CCNTs can contribute effectively to avoid inappropriate ED attendances and emergency admissions and/or facilitate early discharge.

## Competing interests

The authors declare that they have no competing interests.

## Authors’ contributions

RGK conducted data collection and analysis, drafted and revised the manuscript. MB conducted data collection and analysis. SK secured funding, conducted data analysis and revised the manuscript. PP secured funding and revised the manuscript. PC secured funding, conducted data analysis, drafted and revised the manuscript. All authors approved the final version.

## Pre-publication history

The pre-publication history for this paper can be accessed here:

http://www.biomedcentral.com/1471-2431/12/101/prepub
